# Responsible AI in mental healthcare: policy directions and stakeholder insights

**DOI:** 10.3389/fpubh.2026.1814039

**Published:** 2026-05-01

**Authors:** Annika M. Schoene, Rafael Mestre, Stuart E. Middleton, Agata Lapedriza, Aynsley Bernard, Matt Lewis, Janelle Lardizabal, Arezoo Movaghar, Kelvin J. Fenelon, Alisa K. Lincoln, Suzanne Gaverich, Danny Scalise II, Gautham Vijay Kumar, Robert F. Leeman

**Affiliations:** 1 Department of Public Health and Health Sciences, Northeastern University, Boston, MA, United States; 2 School of Electronics and Computer Science, University of Southampton, Southampton, United Kingdom; 3 Institute for Experiential AI and Khoury College of Computer Science, Northeastern University, Boston, MA, United States; 4 Kooth Digital Health Limited, London, United Kingdom; 5 LLMental, A Public Benefit Limited Liability Corporation, New York, NY, United States; 6 Institute for Health Equity and Social Justice Research, Northeastern University, Boston, MA, United States; 7 Department of Pediatrics, Wake Forest University School of Medicine, Winston-Salem, NC, United States; 8 Center for Artificial Intelligence Research, Wake Forest University School of Medicine, Winston-Salem, NC, United States; 9 Department of Public Health, Burke County, Morganton, NC, United States; 10 Knight School of Computing and Information Sciences, Florida International University, Miami, FL, United States

**Keywords:** AI governance, AI in public health, digital mental health, mental healthcare, responsible AI, stakeholder engagement

## Abstract

As artificial intelligence (AI) becomes increasingly integrated into mental healthcare, it presents both opportunities to expand access and challenges related to ethics, safety, and regulation. This white paper examines the current U.S. regulatory landscape and presents findings from a scenario-based survey conducted during the 2025 *Responsible AI for Mental Health* workshop in Charlotte, North Carolina in April 2025. While state efforts are driving more structured oversight, including transparency and clinician accountability, stakeholders remain concerned about risks related to privacy, bias, and inappropriate use. Recommendations emphasize the need for robust human oversight, informed consent, and inclusive design. The paper concludes that responsible AI deployment in mental health requires coordinated policy action, ongoing evaluation, and a strong ethical foundation.

## 1 Introduction

In the United States, more than one in five adults experienced a mental illness in the past year, and approximately one in 20 faced a serious mental illness, such as bipolar or major depression, that substantially impacted everyday life activities (1, 2). Among adolescents aged 12–17, 15% live with depression, and nearly 20% report recent symptoms of anxiety, often without accessing any professional care (3). The largest challenges in delivering mental healthcare include critical shortages of qualified mental health professionals, especially in rural areas, gaps in insurance coverage or provider networks, and rising rates of untreated conditions and suicide, despite growing public awareness (4–8). While 90% of Americans believe there is currently a mental health crisis in the United States, in a 2022 survey, about 1/3 of participants reported lack of access to mental healthcare. High cost, stigma and shame are routinely cited as barriers to care. Relatedly, 60% of psychologists in a 2022 practitioner survey reported no openings for new patients (9, 10). Artificial intelligence (AI) is increasingly viewed as a practical solution to these and other long-standing barriers in providing mental healthcare across the United States and globally. For example, in rural communities over 60% of counties lack a single psychiatrist and patients often face long travel distances, provider scarcity, and limited continuity of care (11). AI-enabled tools could help mitigate these challenges by offering remote screening, automated triage, and digital therapeutic support that reduce dependence on physical infrastructure and specialist availability (12). Furthermore, AI-driven systems have shown promise in supporting diagnosis and care management in settings where human resources are insufficient, particularly in underserved or underinsured populations (13). As many mental health providers operate outside insurance networks due to low reimbursement rates, AI tools could expand access by delivering low-cost, scalable interventions for those who might otherwise receive no care (14). At the same time, projects and investments in the global mental health market are expected to grow to approximately $5 billion by 2030, signaling a shift in how AI is seen as more than a technological novelty, but as a necessary tool that addresses critical gaps (15). However, there are several critical ethical and safety concerns for the use of AI in delivering mental healthcare, including but not limited to:

- Risk of psychological harm as AI interactions may reinforce delusions or emotional dependencies (16, 17), especially for vulnerable users;
- Algorithmic bias that may increase existing disparities due to the use of non-representative data in diagnosis, symptom classification, and treatment recommendations (18, 19);
- The use of sensitive patient data in AI applications that raise concerns around privacy, dual use, and misuse (20);
- A lack of transparency and explainability in how AI systems make decisions creating challenges around provenance, accountability, informed consent, and possibility undermining clinical trust (21, 22).

To navigate these opportunities and risks, it is essential to examine the existing U.S. policy and regulatory landscape surrounding AI in mental healthcare and develop recommendations that can guide the safe and ethical development of new AI-based technologies in this space. In this white paper, we report and analyze the findings of the second iteration of the *Responsible AI for Mental Health* workshop series held in Charlotte (North Carolina) in April 2025. We first present a brief overview of the current policy and regulatory environment for AI in mental healthcare in the United States. Next, we report findings from a survey conducted among stakeholders in the field, highlights resulting recommendations, and discusses the implications of these findings for future research, practice, and policy development.

## 2 Existing U.S. policies and regulations governing AI in mental healthcare

As of 2025, the United States relies on existing laws and agency oversight rather than an AI-specific statute^1^ on a federal level. While multiple bills have been proposed in Congress addressing AI, none focused specifically on mental health or have become law. Instead, the federal approach has emphasized voluntary guidelines and frameworks to avoid perceived barriers to innovation.^2^ Previously entered but now revoked executive orders have broadly outlined standards around AI safety, civil rights, and transparency.^3^ Recent changes reflect an ongoing federal debate between prioritizing innovation and imposing safeguards. One notable development is a provision that was proposed in 2025 budget negotiations to preempt state AI regulations with a *10-year moratorium* blocking states from enforcing their own AI laws.^4^

With limited federal regulation, U.S. states have taken the lead in shaping legal and ethical boundaries, where more than 30 states have proposed or passed AI-related health legislation, many focusing on transparency, consumer protection, and human oversight:^5^

- California has enacted two key laws. Assembly Bill 3030 mandates disclosure when generative AI is used in patient communication, unless reviewed by a licensed provider^6^ and Senate Bill 1120 prohibits health plans from using AI to make medical necessity decisions without human clinical input.^7^
- Utah has implemented SB 149 (requiring AI use disclosure) and HB 452 (the first U.S. law to regulate mental health chatbots). The law enforces transparency, prohibits the sale of user data, bans deceptive advertising, and mandates oversight by licensed professionals.^8^
- Illinois has banned fully autonomous AI therapy through the *Wellness and Oversight for Psychological Resources Act*, reaffirming that only licensed professionals may deliver mental health.^9^
- Massachusetts and Rhode Island have introduced bills requiring licensure board approval for AI use in therapy, informed consent, and continuous human oversight. These proposals emphasize AI as an adjunct for clinical care.^10^
- Colorado passed the AI Accountability Act (SB 205),^11^ requiring bias mitigation and algorithmic transparency across sectors, including health.
- New York has mandated state agency oversight of AI, including within health departments, via Technology Law §103-E.^12^
- Other states (e.g., Texas^13^ and Maryland^14^) have debated or passed legislation addressing AI mental health applications, often targeting false advertising, school-based interventions, or unlicensed practice.

Generally speaking, state-level policies increasingly require disclosure, prevent fully automated decision-making in mental health, and reinforce clinician accountability. These efforts reflect a trend toward embedding ethical and safety principles into AI deployment at the point of care.

Overall, although regulatory efforts remain decentralized across federal and state levels, recent state-level changes could indicate that the policy direction is converging toward more structured oversight with an emphasis on safety audits, mandatory disclosures, and the continued involvement of qualified clinicians. As this landscape evolves, the governance of AI in mental health will likely be shaped not only by statutory and regulatory frameworks but also by professional standards and ethical best practices.

## 3 Stakeholder perspectives: survey findings

### 3.1 Workshop overview

The Responsible AI for Mental Health Initiative (23) brings together researchers, clinicians, ethicists, policymakers, and technology professionals to examine how AI can be developed and deployed responsibly within mental health contexts. The US workshop's central objective was to articulate actionable guidelines for the ethical, fair, and transparent use of AI technologies in mental health. Participants explored the interplay between technical innovation and human wellbeing, emphasizing how AI systems must protect privacy, mitigate bias, and align with principles of clinical safety and psychological trust. Discussions spanned a wide range of themes. Sessions addressed the design of ethical ecosystems for AI and big data; the role of responsible innovation in supporting youth mental health; and approaches for developing scalable and trustworthy digital interventions. Technical panels examined the use of synthetic data to reduce risks associated with large language models (LLMs) and the ethical handling of clinical data such as therapy notes and diagnostic records. The program was structured as a series of keynote talks, thematic panels, and collaborative breakout sessions. These formats facilitated dialogue across disciplines, encouraging the co-creation of principles and practices that balance the potential of AI with the moral and professional duties of mental healthcare.

### 3.2 Survey design and scenario framework

To gather informed stakeholder perspectives on the ethical, regulatory, and implementation challenges associated with the use of AI in mental healthcare, we conducted a survey during the workshop. The survey was distributed to participants representing a range of professional sectors, including academia, digital health, public health, and technology development.

The survey instrument was designed to elicit both professional background information and scenario-based reflections. Participants were first asked to provide their current field of work and role(s), and to indicate their willingness to be listed as a contributor to this white paper. They were also asked whether they consented to being contacted for future feedback on the aggregated content.

At the core of the survey, participants were asked to engage with one of several real-world scenarios involving the use of AI in mental healthcare. Each participants selected one of the following predefined scenarios or contributed a custom scenario from their domain of expertise:

- AI in a public health or educational setting: a school district implements an AI-enabled monitoring system that analyzes student attendance data, health records, and writing samples for signs of mental health distress, such as depression or suicidal ideation. The system alerts school counselors or authorities if risk is detected.
- AI in a clinical setting: a hospital system deploys an AI tool to support psychiatric diagnosis and care planning by analyzing medical histories, genetic data, and responses to clinical assessments. The system generates diagnostic suggestions and potential treatment options to assist psychiatrists.
- AI in an industry setting: a large employer introduces an AI-based monitoring tool that analyzes internal communications (e.g., emails, chat messages) and productivity data to identify potential signs of stress, burnout, or declining mental health among employees. Individuals flagged by the system receive automated wellness recommendations or are referred to internal support resources.

For each selected scenario, participants were asked a series of open-ended questions to identify potential benefits, risks, ethical considerations, implementation challenges, and proposed solutions. Participants were also invited to reflect broadly on other promising applications of AI in mental healthcare, and to offer any final comments or recommendations.

### 3.3 Survey findings

Fourteen individuals participated in the survey component of the study. The most frequently selected scenarios were the use of AI in workplace and educational settings. These scenarios focused respectively on employee mental health monitoring through workplace communication tools and the use of AI-enabled systems in schools to detect student distress or suicidality. Few participants selected the clinical setting scenario, which involved AI-assisted psychiatric diagnosis and treatment planning. In addition to the predefined options, two participants proposed custom scenarios:

The survey participants selected the most frequently scenarios were the use of AI in workplace settings and educational settings. These scenarios focused respectively on employee mental health monitoring through workplace communication tools and the use of AI-enabled systems in schools to detect student distress or suicidality. Few participants selected the clinical setting scenario, which involved AI-assisted psychiatric diagnosis and treatment planning. In addition to the predefined options, two participants proposed custom scenarios:

- One described a comprehensive, AI-supported mental health system for individuals with treatment-resistant depression, emphasizing recovery-oriented care across the continuum from early intervention to relapse prevention.
- Another response addressed AI and ethics more broadly, without aligning neatly with a specific use case.

Across these responses, several convergent themes, scenario-specific concerns, and critical tensions emerged. Below we outline key themes:

- Early detection and system efficiency: across scenarios, participants cited early detection and triage of mental health concerns as potential strengths of AI-based systems, particularly in environments with limited access to mental health professionals. Several participants viewed AI as a scalable complement to overstretched systems, capable of flagging distress signals, supporting decision-making, and directing users toward appropriate interventions. AI was also described as a possible tool for personalizing mental healthcare and improving continuity in settings where individuals might otherwise receive fragmented or no support. These benefits were frequently framed as conditional:

“*AI could help identify patterns that might otherwise be missed, but only if it's implemented with transparency and human oversight*.”

- Privacy, consent, and surveillance risks: privacy and consent were dominant ethical concerns across all scenarios. In educational settings, concerns centered on the use of school-issued devices to monitor student behavior without adequate disclosure to students or families. In workplace scenarios, participants warned that AI systems could be used to infer mental health status from communication data, raising the risk of disciplinary misuse or reputational harm. One participant noted,

“*There is a fine line between offering support and creating a surveillance environment that discourages honesty or help-seeking*.”

- Scenario-specific distinctions: depending on different scenarios, responses revealed important contextual differences in how participants assessed the risks, ethical considerations, and implementation challenges associated with AI use in mental healthcare:

- *Public school* settings raised particular concerns about student autonomy, consent from guardians, and cultural misinterpretation of language or behavior. Participants emphasized that misidentifying distress could lead to stigmatization or disciplinary responses, especially for students from racial or linguistic minority backgrounds.
- *Workplace* settings drew attention to the risk of violating labor protections, including the Americans with Disabilities Act^15^ (ADA), if AI systems were used to infer diagnoses or mental health risk. Multiple participants questioned whether employees could realistically opt out of such programs without penalty or opt-in without stigma.
- *Clinical settings* were viewed more favorably overall, though concerns remained around over-medicalization, lack of attention to social determinants of mental health, and the risk of deskilling clinicians by over-relying on algorithmic outputs.

- Role-based variations in perspective: participants working in industry tended to emphasize usability, implementation feasibility, and scaling. While recognizing ethical risks, these participants often proposed technical solutions such as opt-in designs, anonymization, or explainability as ways to mitigate harm. Those from academic and public health backgrounds focused more heavily on equity, bias, and social context. These participants were more likely to question the structural assumptions behind AI tools and to argue for stakeholder co-design, inclusion of marginalized voices, and the incorporation of non-biomedical frameworks into system design.
- Governance and human oversight: there was consensus that AI systems used in mental health contexts must include some form of human oversight. Suggested approaches included human-in-the-loop decision processes, ethics review committees, and clearer boundaries between administrative and clinical roles (e.g., separating HR oversight from mental health triage). Participants also emphasized that this would necessitate upskilling and training for individuals working with or alongside AI. Reasons stated for human oversight of AI included avoidance of harms, litigation and provision of more patient centric responses. Several participants emphasized that transparency about how data is collected, processed, and used is essential for building trust and accountability. However, few responses elaborated on how such governance structures would be maintained or enforced.
- AI as supportive, not substitutive: across roles, participants cautioned against deploying AI as a replacement for human relationships in mental healthcare. While automation could reduce burden and expand access, participants stressed that therapeutic and supportive interactions rely on context, empathy, and trust which are difficult to replicate algorithmically. There was also a shared concern that in scaling AI tools too aggressively, institutions might neglect the relational and structural dimensions of mental healthcare.

### 3.4 Participants recommendations

Survey participants offered a range of practical and ethical recommendations aimed at guiding the responsible development and deployment of AI in mental health contexts. A primary recommendation was the need for explicit, opt-in consent mechanisms. Participants emphasized that users, specifically students and employees, must have full control over whether and how their data is used in AI systems. Consent must be informed and voluntary, with clear opportunities to opt out without penalty. Users should also be given the ability to access, understand, and, where appropriate, contest or revise the outputs generated about them by AI systems.

Participants also consistently called for robust human oversight in any deployment of AI tools that may flag individuals for potential risk. This included integrating human-in-the-loop workflows and establishing formal oversight structures such as institutional review boards, ethics committees, or cross-sectoral governance bodies. These structures would ensure that flagged cases are reviewed contextually, reduce the likelihood of false positives or misinterpretation, and increase public trust in such systems. A strong emphasis was placed on the need to prioritize equity and cultural responsiveness in system design. Participants recommended developing tools using representative data sets that account for social determinants of mental health and incorporating input from historically marginalized communities. Multiple responses highlighted the risks of over-relying on clinical or biomedical frameworks that do not reflect the lived realities of many populations. Equity-focused design also included addressing language accessibility and cultural nuance in both system behavior and user interaction. To mitigate the risk of function creep and misuse, participants advised implementing technical and organizational safeguards that prevent AI-generated data from being repurposed beyond its original intent. This was particularly important in workplace scenarios, where data collected for mental health support could potentially be accessed by human resources or used for disciplinary action.

Recommended safeguards included establishing clear data boundaries, anonymizing sensitive outputs, and ensuring strict separation between support services and administrative oversight. Transparency and explainability were also recurring priorities. Participants stressed that both end-users and decision-makers should be able to understand how the system reaches its conclusions, the provenance of data used to generate conclusions, and an understandable characterization of the model's limitations. This also included a call for explainable AI methods appropriate for non-technical audiences as well as transparency in how the systems are trained, validated, and maintained over time. However, from a technological standpoint, true explainability in LLMs remains doubtful: models simple enough to be interpretable, such as single-layer neural networks, rarely achieve acceptable performance for complex tasks. For the recent generation of LLMs, approaches like model data cards that clearly outlining limitations, contexts of appropriate use, and domain-specific constraints (e.g., in mental health applications) can offer a more pragmatic balance between explainability and performance.

Finally, participants emphasized the importance of inclusive co-design processes, which included involving stakeholders with lived experience of mental health challenges in all stages of development: from problem definition to implementation and evaluation. Participants also encouraged the development of tools that support peer-led recovery or self-directed care, particularly in communities where traditional clinical services are inaccessible or underutilized. Overall, these recommendations reflect a strong preference for systems that are participatory, accountable, equitable, and constrained in scope. Participants demonstrated an awareness that even well-intentioned AI tools could produce harm without explicit attention to the contexts in which they are deployed and the people they aim to serve.

## 4 Discussion

The recommendations offered by survey Participants reflect a clear need for to responsible, human-centered AI development in mental health contexts and recommendations aligned with existing principles in responsible AI and digital health ethics and highlight the need for proactive safeguards in contexts where power asymmetries, stigma, and vulnerability are acute. Building off the recommendations offered by workshop participants there is a need for further guidance, research, and cross-sector collaboration. This includes structural, regulatory, and operational dimensions that are essential to ensuring not just safe design, but safe deployment and sustained impact:

- There is a pressing need to establish clear accountability and enforcement mechanisms. Ethical principles such as transparency and oversight must be accompanied by systems for monitoring compliance, investigating harms, and offering users channels for recourse. This includes data cards explaining AI model limitations, formal auditing and assurance procedures, reporting requirements, and independent review processes to ensure that AI tools do not silently introduce new forms of risk or discrimination.
- Effective deployment will depend on interoperability and systems integration. AI tools must be designed to interface reliably with existing infrastructures (e.g., electronic health records, student information systems, or employer support programs) to avoid duplication, vendor lock-in, fragmentation, or operational failure, where implementation strategies should address not only technological compatibility but also governance alignment across sectors.
- Regulatory clarity remains under-developed, and it is not yet clear how AI-enabled mental health tools will be classified under existing legal frameworks, or what specific oversight bodies (e.g., FDA or state health departments) will play a role in review and enforcement. Policymakers should consider developing targeted guidance for mental health applications, including distinctions between clinical and non-clinical use cases.
- Economic models for sustainable adoption are largely absent from current discourse. Without reimbursement pathways, public funding mechanisms, or cost-sharing strategies, AI tools may remain inaccessible to under-resourced settings or fall into cycles of short-term implementation and abandonment. Sustainable financing will be particularly important in public systems such as schools, community clinics, or Medicaid-funded programs.
- There is a need to invest in AI literacy training and capacity-building for the individuals expected to interpret, implement, or supervise these tools. Clinicians, educators, and organizational leaders must be equipped not only with technical knowledge, but also with ethical and legal literacy to navigate AI-supported environments. Similarly, individuals subject to monitoring or triage should be informed of their rights, options, and the nature of algorithmic decision-making.
- AI tools for mental health require ongoing evaluation and iterative refinement beyond initial deployment. This includes mechanisms for real-time monitoring, long-term outcome assessment, and structured feedback loops involving users and frontline professionals. Static validation methods are unlikely to capture the evolving risks and inequities that may emerge as tools are scaled or adapted across different populations.

Together, these areas represent necessary extensions to the current discourse on responsible AI and shift the focus from design-phase ethics to full lifecycle governance. Addressing them will be essential to ensuring that AI systems in mental health not only avoid harm, but deliver meaningful, equitable, and sustained benefit. However, the current sociotechnical and policy infrastructure in the United States is in many instances unprepared to ensure the ethical, safe, and equitable deployment of these technologies. By contrast, jurisdictions such as the European Union are building more comprehensive AI regimes (e.g., the EU AI Act) that impose mandatory risk-based assessments, obligations for high-risk systems, transparency requirements, and stronger enforcement mechanisms (24). The lack of systematic regulation, evidence-based evaluation standards, and enforceable accountability mechanisms has created a gap in governance, allowing experimental or unvalidated tools to enter clinical and quasi-clinical settings without appropriate oversight.

## 5 Conclusion and limitation

This white paper examines the evolving regulatory environment surrounding the use of AI in mental healthcare within the United States and reports empirical insights derived from a scenario-based stakeholder survey. The analysis suggests that, while federal regulatory approaches remain fragmented and largely advisory, emerging state-level initiatives are beginning to establish more formalized frameworks that prioritize safety, transparency, and human oversight. The findings underscore a shared recognition among stakeholders that AI should function as a complement to human judgement in mental health contexts, and that systems must be designed with cultural responsiveness, user agency, and robust ethical safeguards.

Despite these contributions, several limitations should be acknowledged. First, the survey sample was limited to attendees of a single workshop, which introduces selection bias. Participants were likely predisposed to interest in responsible AI practices and may not represent the full diversity of viewpoints within the broader mental health, technology, or policy communities. Furthermore, while the scenario-based methodology enabled rich, context-sensitive engagement, the limited number of participants per scenario restricted the breadth of comparative analysis. Finally, the policy overview reflects legislative and regulatory developments as of 2025. Given the rapid pace of technological innovation and policy change in this domain, some of the specific legal or institutional references may have evolved by the time of publication.

## Footnotes

1
https://www.whitecase.com/insight-our-thinking/ai-watch-global-regulatory-tracker-united-states#:~:text=Laws%2FRegulations%20directly%20regulating%20AI%20,%E2%80%9CAI%20Regulations%E2%80%9D

2
https://www.whitehouse.gov/presidential-actions/2025/07/preventing-woke-ai-in-the-federal-government/

3
https://www.beckersbehavioralhealth.com/ai-2/5-bills-targeting-ais-role-in-mental-health/

4
https://www.ama-assn.org/practice-management/digital-health/states-are-stepping-health-ai-regulation#:~:text=regulation%20or%20more%20appropriate%20regulation,the%20AI%20space%2C%E2%80%9D%20Curtis%20said

5
https://www.ama-assn.org/

6
https://leginfo.legislature.ca.gov/faces/billNavClient.xhtml?bill_id=202320240AB3030

7
https://leginfo.legislature.ca.gov/faces/billNavClient.xhtml?bill_id=202320240SB1120

8
https://le.utah.gov/~2025/bills/static/HB0452.html

9
https://www.ilga.gov/documents/legislation/104/HB/PDF/10400HB1806lv.pdf

10
https://le.utah.gov/Session/2025/bills/introduced/HB0452.pdf

11
https://leg.colorado.gov/bills/sb24-205

12
https://www.nysenate.gov/legislation/laws/STT/103-E

13
https://capitol.texas.gov/tlodocs/89R/billtext/html/HB01265I.htm

14
https://mgaleg.maryland.gov/mgawebsite/Legislation/Details/sb0987?ys=2025RS

15
https://www.ada.gov

## References

[1] NIMH. Mental Illness. Bethesda, MD: National Institute of Mental Health (2023). Available online at: https://www.nimh.nih.gov/health/statistics/mental-illness (Accessed June, 2025).

[2] SAMHSA. Results from the 2022 National Survey on Drug Use and Health. Rockville, MD: Substance Abuse and Mental Health Services Administration (2023). Available online at: https://www.samhsa.gov/data/sites/default/files/reports/rpt42731/2022-nsduh-annual-national-web-110923/2022-nsduh-nnr.htm (Accessed June, 2025).

[3] CDC. About Mental Health. Atlanta, GA: Centers for Disease Control and Prevention (2025). Available online at: https://www.cdc.gov/mental-health/about/index.html (Accessed June, 2025).

[4] Time. America has reached peak therapy. Why is our mental health getting worse? Time Magazine (2023). Available online at: https://time.com/6308096/therapy-mental-health-worse-us/ (Accessed August, 2025).

[5] Graham AK Weissman RS Mohr DC. Resolving key barriers to advancing mental health equity in rural communities using digital mental health interventions. JAMA Health Forum. (2021) 2:e211149. doi: 10.1001/jamahealthforum.2021.1149 36218752 PMC11650711

[6] Graves JM Abshire DA Koontz E Mackelprang JL. Identifying challenges and solutions for improving access to mental health services for rural youth: insights from adult community members. Int J Environ Res Public Health. (2024) 21:725. doi: 10.3390/ijerph21060725 38928971 PMC11203972

[7] McGinty BETH. The future of public mental health: challenges and opportunities. Milbank Q. (2023) 101(Suppl. 1):532. doi: 10.1111/1468-0009.12622 37096616 PMC10126977

[8] Health Resources and Services Administration (HRSA). State of the Behavioral Health Workforce Report 2024. Washington, DC: U.S. Department of Health and Human Services (2024). Available online at: https://bhw.hrsa.gov/sites/default/files/bureau-health-workforce/state-of-the-behavioral-health-workforce-report-2024.pdf (Accessed August 6, 2025).

[9] Lopes L Kirzinger A Sparks G Stokes M Brodie M. KFF/CNN Mental Health in America Survey. San Francisco, CA: Kaiser Family Foundation (2022). Available online at: https://www.kff.org/mental-health/kff-cnn-mental-health-in-america-survey/#2a51a1e8-c1f5-43ff-b2f9-4f7a9c4fa802 (Accessed October 7, 2025).

[10] American Psychological Association. Psychologists Struggle to Meet Demand Amid Mental Health Crisis. Washington, DC: American Psychological Association (2022).

[11] Henning-Smith C Fritsma T Olson AP Woldegerima S MacDougall H. Decisions to practice in rural areas among mental health care professionals. JAMA Network Open. (2024) 7:e2421285. doi: 10.1001/jamanetworkopen.2024.21285 38884999 PMC11184455

[12] BHMPC. Technology and the Behavioral Health Shortage. Tampa, FL: Behavioral Health Management and Policy Consultants (2025). Available online at: https://le.utah.gov/Session/2025/bills/introduced/HB0452.pdf (Accessed August 6, 2025).

[13] Babu A Joseph AP. Artificial intelligence in mental healthcare: transformative potential vs. the necessity of human interaction. Front Psychol. (2024) 15:1378904. doi: 10.3389/fpsyg.2024.1378904 39742049 PMC11687125

[14] Kearns WR Bertram J Divina M Kemp L Wang Y Marin A . Bridging the skills gap: evaluating an AI-assisted provider platform to support care providers with empathetic delivery of protocolized therapy. AMIA Annu Symp Proc. (2024) 2023:436. 38222441 PMC10785887

[15] Grand View Research. AI in Mental Health Market Size, Share & Trends Analysis Report By Component (Software, Services), By Technology (Machine Learning, Natural Language Processing), By Region, and Segment Forecasts, 2023 – 2030 (2024). Available online at: https://www.grandviewresearch.com/industry-analysis/ai-mental-health-market-report (Accessed August 6, 2025).

[16] Tavory T. Regulating AI in mental health: ethics of care perspective. JMIR Mental Health. (2024) 11:e58493. doi: 10.2196/58493 39298759 PMC11450345

[17] Patel H Hussain F. Do AI chatbots incite harmful behaviours in mental health patients? BJPsych Open. (2024) 10:S70–1. doi: 10.1192/bjo.2024.225

[18] Ning Y Teixayavong S Shang Y Savulescu J Nagaraj V Miao D . Generative artificial intelligence and ethical considerations in health care: a scoping review and ethics checklist. Lancet Digit Health. (2024) 6:e848–56. doi: 10.1016/S2589-7500(24)00143-2 39294061 PMC11542614

[19] Rahsepar Meadi M Sillekens T Metselaar S van Balkom A Bernstein J Batelaan N. Exploring the ethical challenges of conversational AI in mental health care: scoping review. JMIR Ment Health. (2025) 12:e60432. doi: 10.2196/60432 39983102 PMC11890142

[20] Wang X Zhou Y Zhou G. The application and ethical implication of generative AI in mental health: systematic review. JMIR Ment Health. (2025) 12:e70610. doi: 10.2196/70610 40577783 PMC12254713

[21] Saeidnia HR Hashemi Fotami SG Lund B Ghiasi N. Ethical considerations in artificial intelligence interventions for mental health and well-being: Ensuring responsible implementation and impact. Soc Sci. (2024) 13:381. doi: 10.3390/socsci13070381

[22] Chustecki M. Benefits and risks of AI in health care: narrative review. Interact J Med Res. (2024) 13:e53616. doi: 10.2196/53616 39556817 PMC11612599

[23] Responsible AI for Mental Health (RAI4MH). Responsible AI for Mental Health Initiative (2025). Available online at: https://rai4mh.com/ (Accessed October 10, 2025).

[24] Middleton SE Letouzé E Hossaini A Chapman A. Trust, regulation, and human-in-the-loop AI: within the European region. Commun ACM. (2022) 65:64–8. doi: 10.1145/3511597

